# Pathological Character of Remittent Fever

**Published:** 1846-09

**Authors:** 


### ECLECTIC DEPARTMENT,

### AND SPIRIT OF THE MEDICAL PERIODICAL PRESS.

---

### *Pathological Character of Remittent Fever.*

The following summary of the pathological appearances in ten subjects dead with remittent fever is taken from an article in the *American Journal of Medical Sciences*, for April, 1846. In the article referred to, the history of the cases is given in full. They occurred at the Baltimore Alms House, the medical treatment being directed by Dr. Robinson, and their histories and dissections recorded by Drs. N. F. Anderson, and Charles Frick. The report of the cases does not present any thing new or particularly interesting, and we therefore omit this portion of the article. The comparison of the cases with regard to the pathological appearances, and the following summary were made by Dr. Alfred Stille, of Philadelphia. The results would seem to confirm the observations previously made and communicated to the profession by Drs. Stewardson, of Philadelphia, and Dr. Swett, of New-York :

The number of cases in this series is not large enough, nor is the story of the symptoms in them minute enough, to serve as the groundwork for a full description of remittent fever. But the cadaveric appearances were so very uniform, and correspond so closely with those described by previous observers, as to require a degree of importance entitling them to separate analysis. We shall not, however, attempt more than to bring together the most important and similar features of the several cases.

*Brain.*—This organ was examined in seven out of the ten cases, and in *all* of them, either its membranes or its substance was found injected, and in two of them there was moderate effusion in the cavity af the arachnoid.

*Lungs.*—In one-half of the cases, the lungs are described as healthy, and in the other half there was more or less intense redness of the bronchia, and, in one case, of the larynx. But this condition does not seem to have been accompanied with cough during life, and its inflammatory nature may therefore well be doubted.

*Heart.*—The heart was examined in nine cases, and in *all* of them its muscular tissue was found to be more or less softened. The only one in which this condition was not remarkable, (Case IX,) also presented large fibrinous concretions in both ventricles. The patient had been "very delirious," and some portions of his pia mater were found "infiltrated with a turbid milky serum."

*Stomach and Intestines.*—The stomach generally contained from two to four ounces of a dirty yellow fluid. The mucous membrane was found to be injected in seven out of the nine cases in which it was examined, and three of them intensely so. In five cases it was softened near the cardiac extremity, and in four near the pylorus, where also it was for the most part grayish, thickened, and mammilated. In every instance Brunner's glands were unusually developed, and in three cases to a remarkable degree. The glands of Peyer were constantly healthy, but generally visible.

*Spleen.*—In *all* cases, without exception, the spleen was very much enlarged, being from two to six times larger than natural. In one instance it weighed three pounds. In nine out of ten cases it was very soft or pulpy, and of a bluish black color.

*Liver.*—The size of the liver was noted in nine cases, in all of which it was unnaturally large. Its consistence was very much diminished in ten cases, in eight of which the right lobe was the principal seat of the alteration; in one the left lobe was chiefly affected, and in the remaining one the whole organ was softened. In *all*, the color of the liver was either bronzed, or like that of slate; the surface of a section was polished or shining; and in every instance but one, the different colors of its component parts could not be distinguished. In seven out of eight cases in which the *gall-bladder* was recorded, this receptacle was *distended* with thick grumous bile, resembling molasses. In the eighth case it was moderately distended with straw-colored bile.

From this summary we may now conclude that the cases of remittent fever under examination presented the following lesions uniformly, to wit: 1st. Congestion of the brain; 2d. Softening of the heart; 3d. Softening of the mucous membrane of the stomach; 4th. Softening of the spleen, with enlargement; 5th, Softening of the liver, with enlargement, and a bronzed or slate-like hue of that organ, and distension of the gall-bladder with inspissated bile.

Of all these morbid alterations, the only one peculiar to remittent fever is that of the liver, which was for the first time pointed out, and so well described by Dr. Stewardson, and which the present series of cases, taken along with those previously observed by himself and by Dr. Swett, justifies him in regarding as the *anatomical characteristic* of the disease. But it does not stand alone. The spleen, the stomach, the heart and the brain, are all diseased, and what is still more remarkable, they, with the liver, have one lesion in common, viz: softening. The hepatic alteration is evidently not that to which the symptoms of remittent fever can be referred as a cause. Our knowledge of the phenomena attending inflammation of the liver on the one hand, and of the close analogy existing between remittent fever and intermittent fever (in which the liver is unchanged,) on the other, forbide such a supposition. The bronzed and slaty hues of this organ are pretty certainly due to the congestion of its biliary ducts with bile, and of its veins with blood, so that its softening only remains to be studied, as well in its origin as in its effects. But this softening, as already remarked, is common to it and to several other organs. The question is therefore enlarged, and we have next to enquire to what ought the diminished consistence of these several organs to be attributed? Here are two parenchymatous structures, (the liver and spleen,) a mucous membrane, (of the stomach,) and a muscle, (the heart,) softened in the same disease. It will not be pretended that the change is due to inflammation; for the symptoms of this condition, as it occurs in the several organs, are wanting. Is it owing to a cause like that which produces softening in typhus fever, and in all diseases of a typhoid type? in one word, is it due to an alteration of the blood?

At this points our facts fail; for in most of the reports of dissections in remittent fever, little or nothing is said of the state of the blood, and but little of the consistence of the solids. Dr. Stewardson, indeed, suggests "that an altered condition of the blood, combined, perhaps, with some softening of the tissue" of the lungs, may have given rise to an effusion of bloody serum noticed by him; he also notes the "flabbiness" of the heart in some instances, and the absence of firm fibrinous coagula in all, and remarks that "it is perfectly evident that the blood in this disease is the seat of morbid changes which deserve attention;" and, again that to the state of this fluid "we must no doubt look, in part, for an explanation of the fatal termination in some cases." These observations and surmises are strengthened by the above series of cases; for in them softening of the liver and heart especially, appears to have been more uniformly observed by Dr. Frick than it had been by either of the gentlemen whose names have been mentioned before. But no certain inference can be made from the materials which now exist. Had observers recorded fully all that their cases presented, instead of so much, only, as appeared to bear upon a single point of their history, we should not now have to leave unanswered the main questions relating to the pathology of remittent fever. We therefore call upon all such as have an opportunity to study this disease, to furnish complete accounts of what they witness, detailing minutely all the symptoms that occur during life, and all the changes detected after death, not contenting themselves with confirming previous observations, but rather endeavoring to add some new truths to those already established. This is a subject of serious interest, for on its proper investigation must depend the lives of hundreds every year. A study of the pathology of remittent fever will not, indeed, lead us *directly* to a successful plan of treating the disorder, but *indirectly* it will so lead us, by showing its resemblance to some class or classes of disease of which the therapeutic management has been settled by experience. It may, it probably will, show us that the malady is not everywhere identical, and it will distinguish the cases to which one, and those to which another treatment is applicable. It will also teach us what are causes, and what effects, in the several links of its morbid chain of symptoms and lesions, a knowledge which, at present, we are very far from possessing. The points to which we beg leave earnestly to direct the attention of observers, are the following: 1st. The precise circumstances of the commencement of an attack of remittent fever, including external relations, and symptoms properly so called. 2d. The state of the blood in the several stages of the disease determined according to the method pursued by Andral and Gavarret, and its condition in the vessels and heart after death. 3d. Chemical analyses of the blood, urine and bile; and 4th. The consistence of the solids, especially of the brain, lungs, heart, liver, spleen and kidneys.

In connection with the above we quote the following from an extended and able essay on Remittent Fever by Dr. Boling, of Montgomery, Alabama, published in the same, and a subsequent number of the Am. Journal. It will be observed that Dr. Boling's observations do not correspond with those of Drs. Stewardson and Swett. We have intended to notice Dr. Boling's paper more fully, and may do so hereafter.

"In an article published by Dr. Stewardson, in the *American Journal of Medical Sciences* for April, 1841, in which, among certain other post-mortem appearances which he is disposed to think somewhat peculiar to remittent fever, he describes an appearance of the liver not heretofore particularly noticed, and, from the result of a good many examinations, arrives at the conclusion that it is probably "the anatomical characteristic of that disease." This alteration is described as consisting, in most cases, in a flabby state of the organ, the color externally being of a bronze or a mixture of bronze and olive, and internally of an olive, with an entire extinction of the natural reddish-brown. It appears to have been generally of the natural size, and "the two substances so blended as to be scarcely distinguishable." The appearances described by Dr. Stewardson, have since been found by others, and are particularly noticed by Dr. Swett in the *American Journal of Medical Sciences* for January, 1845. I have given above what, on a pretty close examination of Dr. Stewardson's cases, seems to me to have been the appearance in a large number of them, but still the description of the "*anatomical characteristic*," as given by himself, differs materially from this in some of the others. For instance, we have one described as being "of a pale, slightly greenish lead color," one "of a dull bronze," and in another "the surface of a dull lead color." In the cases of Dr. Swett there is also found this diversity of appearance in the color of the liver. One case "presented externally a slate color, which, when placed in a proper light, gave a bronze tint." Another was of "a pale slate color, and in many parts a bronze tint was perceptible,"—and another "externally was of a bronzed appearance."

On the appearance of the above article of Dr. Stewardson, my attention was again directed to the liver, but I have been able in but a very few instances to find any alteration; and even where such did exist, a slight exertion of the imagination would have, perhaps, been necessary to trace a close resemblance between them and the descriptions of Dr. Stewardson. Indeed, in a large proportion of cases, the organ, so far as I am capable of judging, was entirely healthy. Where it was otherwise, the *concave* surface of the organ was of what appeared to me an uniformly bluish-slate color, extending to about the depth of a quarter of an inch; the line of demarkation between this, and that portion which presented a natural appearance, being well defined. In every case in which I have observed this appearance, with once exception it was confined to the concave surface, the convex surface and the interior being entirely healthy. The exception was in a case which, in the beginning, presented the symptoms of what is generally denominated verminous or infantile remittent fever. In its progress peritonitis supervened, and the patient died about the beginning of the fourth week. On a post-mortem examination, a considerable quantity of serum and coagulable lymph was found in the abdomen, and *both* surfaces of the liver were found uniformly of the color above alluded to, the interior of the organ being apparently healthy. This appearance of the liver I have observed in an equal proportion of cases, I think, in the post-mortem examination of patients dying from other diseases; though I have no recollection at this time of having observed it in any case, in which the patient had not labored under an attack of remittent or intermittent fever, within a month or six weeks previous to the commencement of the disease causing death.

In conclusion, I would observe, that all my remarks in regard to the symptoms, treatment, and post-mortem appearances of remittent fever, are made with reference to the disease as it prevails here in Montgomery, and in its immediate vicinity, for, as I have before observed, these may all vary in different localities. "*Romæ scribo et in Ære romano.*"

